# All Solution-processed Stable White Quantum Dot Light-emitting Diodes with Hybrid ZnO@TiO_2_ as Blue Emitters

**DOI:** 10.1038/srep04085

**Published:** 2014-02-13

**Authors:** Jing Chen, Dewei Zhao, Chi Li, Feng Xu, Wei Lei, Litao Sun, Arokia Nathan, Xiao Wei Sun

**Affiliations:** 1 School of Electronic Science and Engineering, Southeast University, Nanjing, China, 210096; 2 Department of Electrical Engineering and Computer Science, the University of Michigan, Ann Arbor, Michigan 48109, USA; 3 Electrical Engineering Division, Engineering Department, University of Cambridge, 9 JJ Thomson Avenue, CB3 0FA, Cambridge, UK; 4 School of Electrical and Electronic Engineering, Nanyang Technological University, Nanyang Avenue, Singapore 639798; 5 These authors contributed equally to this work.

## Abstract

White quantum dot light-emitting diodes (QD-LEDs) have been a promising candidate for high-efficiency and color-saturated displays. However, it is challenging to integrate various QD emitters into one device and also to obtain efficient blue QDs. Here, we report a simply solution-processed white QD-LED using a hybrid ZnO@TiO_2_ as electron injection layer and ZnCdSeS QD emitters. The white emission is obtained by integrating the yellow emission from QD emitters and the blue emission generated from hybrid ZnO@TiO_2_ layer. We show that the performance of white QD-LEDs can be adjusted by controlling the driving force for hole transport and electroluminescence recombination region via varying the thickness of hole transport layer. The device is demonstrated with a maximum luminance of 730 cd/m^2^ and power efficiency of 1.7 lm/W, exhibiting the Commission Internationale de l'Enclairage (CIE) coordinates of (0.33, 0.33). The unencapsulated white QD-LED has a long lifetime of 96 h at its initial luminance of 730 cd/m^2^, primarily due to the fact that the device with hybrid ZnO@TiO_2_ has low leakage current and is insensitive to the oxygen and the moisture. These results indicate that hybrid ZnO@TiO_2_ provides an alternate and effective approach to achieve high-performance white QD-LEDs and also other optoelectronic devices.

Quantum dot LEDs (QD-LEDs) have been attracting much attention in the past few years since they possess unique properties of the tunable emission wavelengths by controlling the size of QDs, highly saturated emission, narrow emission with small full width at half maxima (FWHM), solution process, and compatible with flexible substrates^1^,^2^,^3^. Especially, the commercialization of white QD-LEDs is expanding to high-volume applications, including general solid-state lighting^4^ and backlight for liquid-crystal displays^5^. White QD-LEDs can achieve a warmly white shade and a good spectral overlap with the sensitivity function of human eye, which in turn increases the efficiency of the light source^6^.

A few strategies have been proposed to achieve white QD-LEDs. Firstly, the architecture of QD-integrated white LEDs is typically fabricated by integrating QD luminophores with different emitting colors on a blue- or near UV-emitting InGaN LED chip. One of the primary advantages of QD-LEDs is the feasibility of being deposited on any type of substrate, including glass and plastics, and therefore they can be further used in light-weight, robust, and rollable displays^1^. However, the advantage may be restricted by applying this structure in white LEDs. Secondly, another prototype results from the direct electroluminescence (EL) of QDs through integrating red, green, and blue emitting QDs to produce high-efficiency white QD-LEDs^7^. However, on one hand, the growth and synthesis of high quantum yield (QY) of deep-blue-emitting QDs (lower than 440 nm) is very rare with low efficiency^8^; on the other hand, it is quite challenging and difficult to stack various QD emitters together^2^. Thirdly, one promising approach is to combine multiple emissions from different recombination regions such as organic charge transport layer, essential QDs layer, and even interfacial exciplex state^9^,^10^.

In order to realize efficient electron injection with vacuum-free process, zinc oxide nanoparticles (ZnO NPs) have been considered as a promising candidate in the development of excitonic optoelectronic devices such as QD-LEDs because of its direct bandgap of 3.3 eV at room temperature and large exciton binding energy of 60 meV^11^. Due to its thermal stability and less sensitivity to oxygen and moisture, ZnO NPs is an ideal alternative to organic electron-transport layer (ETL) employed in QD-LED devices. In addition, ZnO has higher electron mobility (μ_e_ = ~1 cm^2^ V^−1^ s^−1^) than organic semiconductors (such as Alq_3_: μ_e_ = ~1.0 × 10^−5^ cm^2^ V^−1^ s^−1^), which facilitates efficient electron transport, and balances the electrons and the holes in the emission region, as well as consequentially increases the rate of charge recombination^2^. Furthermore, as an inorganic transport material, ZnO has good optical features due to its high refractive index and better wave guiding^12^, which is helpful for the enhancement of external quantum efficiency (EQE) of the QD-LEDs. Therefore, it is demonstrated that ZnO, used as an electron injection layer, benefits for the device stability, the ease of fabrication, and the possibility of high-throughput manufacturing^2^. It has been reported that ZnO layer facilitates the interfacial charge transfer that improves the emission stability of QD emitters, resulting in an EQE close to the theoretical maximum of 20%^13^. In addition, it is interesting that blue emissions can be found from photoluminescence (PL) of ZnO NPs due to the interstitial-zinc-related defect in ZnO^14^. Hence, it provides a new methodology to design and realize a white QD-LED with ZnO NPs as a blue emitter.

Herein, we report the design and fabrication process of white QD-LED with hybrid ZnO NPs/TiO_2_ layer as ETL, in which ZnO NPs also work as a blue emitter. As an alternative to blue LED chip, the blue emission resulting from ZnO NPs is integrated with the yellow emission from QDs emitter by a simple solution process to generate white light. Our best device achieves a maximum luminance of 730 cd/m^2^ and power efficiency of 1.7 lm/W. To our knowledge, it is the first report to make use of ZnO NPs as blue emitters to obtain a white QD-LED.

## Results

**Figure 1:**
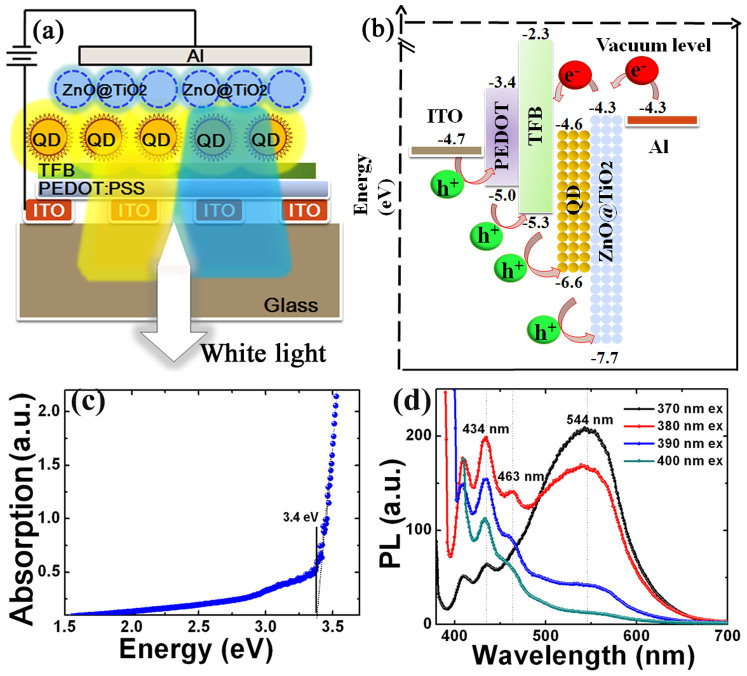
(a) A schematic of the device structure and (b) the corresponding energy band diagram of a white QD-LED. (c) UV-Vis absorption spectrum of hybrid ZnO@TiO_2_ film (d) PL spectra of hybrid ZnO@TiO_2_ dispersed in butanol under different excitation light from 370 nm to 400 nm.

The schematics of our device structure and corresponding energy level diagram are shown in Fig. 1a and b. The device consists of a patterned ITO as the anode, a 50 nm poly(ethylenedioxythiophene):polystyrene sulphonate (PEDOT:PSS) as the hole injection layer (HIL), a variable (10~40 nm) poly[(9,9-dioctylfluorenyl-2,7-diyl)-co-(4,4-(N-(4-sec-butylphenyl)) diphenylamine)] (TFB) layer as the HTL, a 30 nm QD layer as the emissive layer with 3 to 4 closely packed QD monolayers, a 40 nm ZnO@TiO_2_ layer as the ETL, and a 100 nm aluminum (Al) layer as the cathode.

The QD-LED structure was designed to achieve efficient electron and hole injection from the electrodes to the QD layer. Meanwhile, it is effectively blocking electrons and holes that pass through the QD layer in terms of the energy levels of the constituent layers (Fig. 1b). A small injection step of 0.3 eV exists for the injection of electrons from Al to the QD layer since the hybrid ZnO@TiO_2_ has an electron affinity of 4.3 eV, similar to the work function of Al (4.3 eV). The small barrier between the highest occupied molecular orbital (HOMO) of PEDOT: PSS and TFB allows facile injection of holes from ITO to the QD layer. It is reported that the barrier for hole injection is much smaller (~0.2–0.3 eV) since the ionization energy of QD was measured to be 1 eV shallower than that derived from the value of ultra violet photoelectron spectroscopy measurement (6.5–6.9 eV)^13^. Therefore, an Auger-assisted energy upconversion existing at the polymer/QD interface contributes to the EL of QD-LEDs at the low driving voltage^15^. Meanwhile, the high lowest unoccupied molecular orbital (LUMO) of TFB and low valence band (VB) of ZnO can effectively block injected electrons and holes, respectively, leading to the confinement of the charges within the QD layer. However, as the driving voltage or internal electric field is high, it is likely for holes to tunnel through the QDs and to accumulate at the interface of QD/ZnO NPs. Fig. 1c and d show the absorption of hybrid ZnO@TiO_2_ film and PL spectra of that dispersed in butanol under different excitation light from 370 nm to 400 nm. From Fig. 1c, the optical bandgap of hybrid ZnO@TiO_2_ is determined as 3.4 eV. The PL spectrum of ZnO NPs shows a green emission located at 544 nm under the excitation of 370 nm light (Fig. 1d). As the excitation light changes from 370 nm to 400 nm, the green emission disappears, and meanwhile the blue emission peak and shoulder located at 434 nm (2.86 eV) and 463 nm (2.68 eV) are present, respectively, originating from the interstitial-zinc-related defect level as initial states^14^.The common green emission of ZnO is only efficient at the condition that ZnO is excited by above bandgap energy, resulting in the transition from the conduction band (CB) to deep levels^16^; while the blue emissions of ZnO is likely attributed to the transition from extended Zn interstitials (Zn_i_) states (~2.9 eV), which are slightly below the simple Zn_i_ state, to the VB. These extended states could be formed during the annealing process due to the defect ionization reaction^14^.

**Figure 2:**
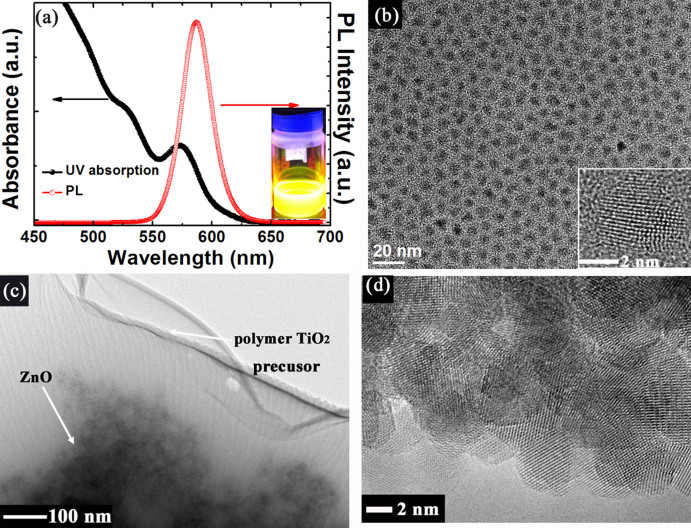
(a) UV-Vis absorption and PL spectra of ZnCdSeS QDs dispersed in toluene. The inset shows the photo of QD solution irradiated by 365 UV light. (b) TEM image of ZnCdSeS QDs. The inset shows the HRTEM image of single ZnCdSeS QD. (c) TEM image of hybrid ZnO@TiO_2_. (d) HRTEM image of ZnO NP.

Figure 2a shows the absorption and PL spectra of ZnCdSeS QDs used in this study. The absorption spectrum clearly displays the first excitonic transition peak at 570 nm, estimating the sizes of these QDs to be 3.2 nm without the ligand length, calculated as reported^17^. The PL spectrum shows a Gaussian-shaped peak located at 586 nm with a narrow FWHM of 30 nm. The inset of Fig. 2a shows the photo of a vial of ZnCdSeS solution exited under 365 nm light, exhibiting yellow emission. The high resolution transmission electron microscope (HRTEM) image, as shown in Fig. 2b, indicates that ZnCdSeS QDs without core-shell structure were uniformly dispersed in toluene with an average diameter of ~5 nm. The QY of ZnCdSeS QDs can be calculated as 64%, using Rhodamine 6G as reference^18^. Unlike core-shell CdSe/ZnS QDs, ZnCdSeS QDs lead to lower confinement of the electron wave-function and high degree of surface accessibility by photo-induced electrons, achieving a relatively high QY^19^. Fig. 2c and d show the TEM and HRTEM images of hybrid ZnO@TiO_2_ which consists of ZnO NPs and underneath TiO_2_ film dispersed in butanol with an average diameter of ~4 nm. It is observed that ZnO@TiO_2_ in butanol has the best stability and dispersibility as well as smallest contact angle on QD film (Supplementary information Fig. S1). The HRTEM image implies good crystallinity of ZnO NPs due to the clear lattice fringe.

After annealing of ZnO@TiO_2_ ETL at 150°C in air, the polymer TiO_2_ precursor is converted to TiO_2_ NPs, whereas pure ZnO film or TiO_2_ film tends to be polycrystalline with pronounced grain boundaries, revealed by atom force microscopy (AFM) images (Supplementary information Fig. S2). The ZnO@TiO_2_ layers are relatively smooth (RMS is lower than 3 nm) and crystallographically amorphous, reducing the likelihood of morphology-induced electrical shorts of the device. Moreover, during the annealing process, organic ligands capping the QDs could be oxidized, producing trap sites that lead to nonradiative recombination of QD excitons^20^. Thus, the performance of QD-LED with hybrid ZnO@TiO_2_ as ETL is anticipated to be enhanced largely.

**Figure 3:**
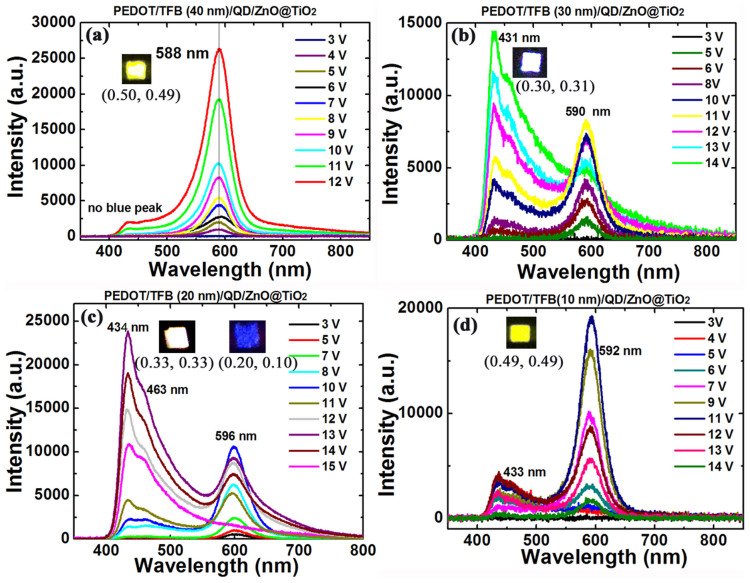
(a)–(d) The EL spectra of sample A-D at the operation voltage varying from 3–15 V. The insets of Figure 3 (a)–(d) show the emission photos for one pixel of sample A-D.

The EL spectra of sample A-D at the driving voltage varying from 3 to 15 V are shown in Fig. 3. In Fig. 3a (TFB thickness is 40 nm for sample A), it can be seen that the EL intensity of 588 nm emission increases until the driving voltage reaches 12 V, showing the Commission Internationale de l'Enclairage (CIE) coordinates of (0.51, 0.49) and the luminance of 400 cd/m^2^. However, the blue emission does not appear until the intensity of yellow emission starts to reduce. As the thickness of TFB decreases to 30 nm (sample B), the blue emission is observed at the driving voltage of 10 V (Fig. 3b). The intensity of blue emission increases, resulting in the white light due to the integration with yellow emission from QDs. The CIE coordinates shift from (0.45, 0.41) to (0.30, 0.31) with the driving voltage changing from 11 to 14 V. The intensity of yellow emission decreases as the driving voltage exceeds 12 V, leading to the low brightness of the white light. When the thickness of TFB is further reduced to 20 nm (sample C), the blue emission is still obvious. It is clear that the CIE coordinates shift from (0.40, 0.38) to (0.33, 0.33) as the driving voltage increases from 11 to 13 V. Beyond 13 V, both intensities of blue and yellow emissions decrease, especially for the yellow emission, exhibiting the only blue emission with CIE coordinates of (0.20, 0.10) at 15 V. The trace emission track on CIE 1931 chromaticity space of sample C under the driving voltage varying from 10–15 V and photos of QD-LED prototypes are shown in Fig. S3 (Supplementary Information). The EL spectra of sample D with 10 nm TFB are presented in Fig. 3d. The FWHM of the yellow emission of sample D is narrower than that of sample A with CIE coordinates of (0.49, 0.49). The blue emission of sample D can be measured; however, its intensity is much lower than that of sample C. Meanwhile, the yellow and blue emissions degrade at the driving voltage of 12 V and 13 V, respectively. Therefore, sample D does not produce white light. Compared to the PL spectrum of QD (Fig. 2a), the slight redshift (5–10 nm) of EL spectrum of the identical QDs might be attributed to the inter-dot interaction, which has been observed previously in closely packed QD solids, and also to the electric-field-induced Stark effect^13^,^21^.

**Figure 4:**
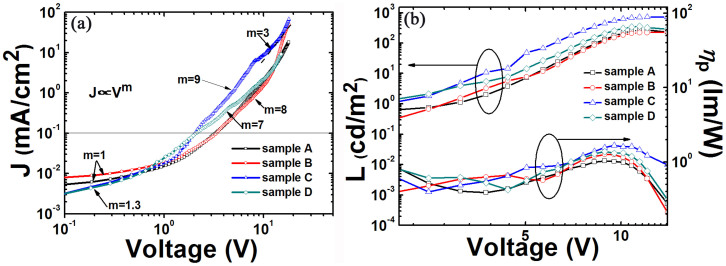
(a) Current density versus driving voltage for sample A to D and (b) Luminance versus driving voltage and Power efficiency versus driving voltage for sample A to D.

All QD-LEDs were tested as-made in air for several days, without encapsulation, and stored at atmosphere between tests. Fig. 4a shows the current density-voltage (J–V) of sample A-D under forward bias. The devices exhibit low turn-on voltage of 2.0 V for sample C and D, and 3.4 V for sample A and B, primarily attributed to the Auger-assisted energy upconversion process in QD-LEDs^2^. Sample C displays ohmic conduction up to 2 V, trap limited conduction (J∝V^n^, n > 2) up to 8 V, followed by pseudo space-charge-limited conduction (J∝V^3^) at higher voltages. The current density of sample C and D in the ohmic conduction region is lower than that of sample A and B, indicating that the leakage current of sample C and D decreases remarkably. The slope of the J-V curve for sample C is steeper than those of sample A and B in the trap-limited conduction region. This is attributed to improved charge transport in sample C beyond the threshold voltage causing higher electric field which is beneficial for hole transport. A space-charge-limited conduction region is distinguishable for sample C, indicating that greater charge accumulation exists at the interface of QD/ZnO NPs. Fig. 4b shows the brightness and power efficiency of sample A-D under the driving voltage varying from 2 to 14 V. The maximum brightness of 730 cd/m^2^ and power efficiency of 1.7 lm/W are achieved for sample C. The maximum power efficiency of sample C is enhanced by 26–60% compared to those of sample A, B, and D.

**Figure 5:**
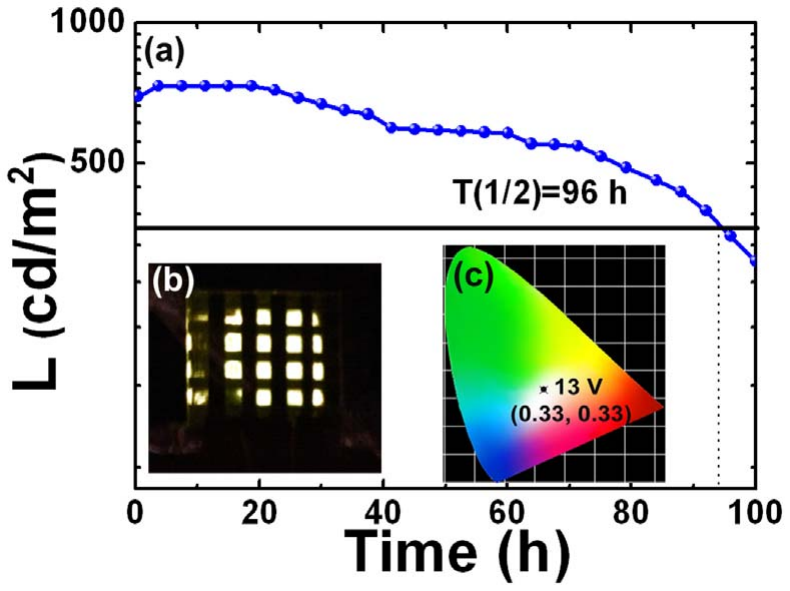
(a) Dependence of luminance for the unencapsulated QD-LED (sample C) at a constant current of 25 mA/cm^2^ on the operation time. (b) A photo of white QD-LED with 4 × 4 pixels at 700 cd/m^2^. (c) CIE coordinates of (0.33, 0.33) for white QD-LED at 13 V.

The stability of the unencapsulated QD-LED (sample C) at 25 mA/cm^2^ is shown in Fig. 5a. Figure 5b and c show the photos of white QD-LED with 4 × 4 pixels at 700 cd/m^2^ and corresponding CIE coordinate of (0.33, 0.33), respectively. As the driving voltage was applied to the QD-LED, the luminance was gradually increased to 730 cd/m^2^ within half an hour, and then it slowly decreased to 365 cd/m^2^ after 96 h. Compared to bare ZnO NPs-based QD-LED shown in Fig. S4 (Supplementary Information), the lifetime of ZnO@TiO_2_-based QD-LED is enhanced, mainly ascribed to the lower current leakage induced by hybrid ZnO@TiO_2_ layer than that by grain boundary of ZnO NPs. In addition, ZnO@TiO_2_ annealed in air as the protect layer prevents the oxygen and moisture from penetrating into the organic layer of QD-LED.

## Discussion

It has been found that the intensity of blue emission is partially dependent on the thickness of HTL. Auger-assisted energy upconversion model has been used to describe the EL process in ZnO NPs based QD-LEDs^2^. Similar mechanism is preferably adopted here that holes and electrons are likely to accumulate at the interface between TFB and the QD EML due to the large energy offsets for both charges at this type-II heterojunction. An Auger-assisted hole injection process can take place, hence the high-energy holes can overcome the injection barrier and recombine with the electrons inside the QDs EML to emit photons. As the driving voltage increases, electrostatic potential caused by hole accumulation at the interface between the TFB layer and the QD layer is enhanced by the stronger driving force^1^. Meanwhile, as the thickness of TFB layer decreases, the electric field in QD-LED is increased, facilitating the transportation of the holes through the QD EML to accumulate at the QD/ZnO@TiO_2_ interface. Due to the interstitial-zinc-related defect emission in ZnO, a mass of electrons up to the extended Zn_i_ energy level can recombine with the holes to generate blue emission at 434 nm, confirmed by PL spectra of ZnO NPs (Fig. 1d). Therefore, the electric field in sample C is higher than those in sample A and B, leading to higher intensity of the blue emission as the driving voltage increases. However, high voltage makes excessive holes and correspondingly higher electric field in the QD layer results in the charging of QDs, which enhances the possibility of non-radiative three body Auger relaxations and decreases EL efficiency^22^. Thus, EL quenching is rapidly obtained in sample D with the increase of the driving voltage.

Compared to the QD-LED based on pure ZnO NPs as ETL, there are several advantages of using ZnO@TiO_2_ as ETL. Firstly, the anti-corrosion and thermal stability of TiO_2_ are higher than those of ZnO, therefore, QD-LED based on ZnO@TiO_2_ has superior stability. Secondly, the roughness of ZnO@TiO_2_ is smaller than that of ZnO as shown in Fig. S2 (Supporting information), leading to lower current leakage in QD-LED device. Thirdly, the bandgap of TiO_2_ is 3.9 eV (CB is −3.9 eV and VB is −7.8 eV), while that of ZnO is 3.3 eV (CB is −4.3 eV, VB is −7.6 eV), i.e. a small energy step between ZnO and TiO_2_, which benefits the electron transfer and leads to lower turnon voltage of QD-LED. Therefore, the overall lifetime of ZnO@TiO_2_ based QD-LED is enhanced.

In summary, we have demonstrated, for the first time, an all solution-processed stable white QD-LED by integrating the yellow emission from ZnCdSeS QD emitters and the blue emission generated from hybrid ZnO@TiO_2_ layer. The mechanism of the blue emissions is proposed to originate from the interstitial-zinc-related defect level as initial states, which is recently observed and proved by PL spectra of ZnO NPs (Fig. 1d). To our knowledge, it is the first time to be found and utilized in EL devices, especially in QD-LEDs. Interestingly, the emission colors of the QD-LEDs can be tunable due to the change of the recombination region with the variance of the HTL thickness (Fig. 3). More importantly, the incorporation of ZnO@TiO_2_ layer improves the lifetime of white QD-LEDs due to the low current leakage and the protection of oxygen and moisture into organic and QDs layer (Fig. 5). Our work offers a promising approach to further develop high-efficiency and stable white QD-LEDs.

## Methods

### Preparation of ZnO@TiO_2_ sol-gel solution

ZnO NPs were prepared by the method as follows. At first, 0.1 M zinc acetate dihydrate (Zn(Ac)_2_·2H_2_O) was dissolved in methanol at room temperature. Then, 0.3 M potassium hydroxide (KOH) in methanol solution was added drop-wise at 40°C with magnetic stirring. The stirring process was kept at 40°C for 1 h at atmosphere. The prepared product was collected by centrifugation and then washed twice with methanol. The white precipitate was re-dispersed in butanol (30 mg/ml) to form ZnO NP solution. A TiO_2_ sol–gel precursor (DuPont tyzol BTP) was diluted to 5 wt% in butanol, followed by being mixed with ZnO NP solution at the volume ratio of 1:1(vol %). Finally, ZnO@TiO_2_ sol-gel solution was ready for usage.

### Preparation of ZnCdSeS QDs

The high-quality yellow emitting ZnCdSeS QDs were synthesized according to a method reported in the literature^23^. As a typical synthetic procedure, 0.4 mmol of CdO, 4 mmol of zinc acetate, 15.5 mmol of oleic acid (OA), and 30 mL of 1-octadecne were placed in a 100 mL round flask. The mixture was heated to 150°C in flowing high-purity N_2_ for 30 min, and further heated to 300°C to form a clear solution of Cd(OA)_2_ and Zn(OA)_2_. At this temperature, a stock solution containing 5 ml of trioctylphosphine, 0.4 mmol of Se, and 4 mmol of S was quickly injected into the reaction flask. After the injection, the reaction temperature was kept for 3 min for promoting the growth of QDs, and then cooled down to room temperature to stop the growth. QDs were washed with acetone for three times, and finally dispersed in toluene at the concentration of 10 mg/ml.

### Device Fabrication

ITO glass substrate with a sheet resistance of about 20 Ω/square was cleaned by successive ultrasonic treatments in detergent, de-ionized water, acetone, and iso-propanol for 15 min, respectively, followed by being treated with ultraviolet light ozone for half an hour. The poly (ethylenedioxythiophene): polystyrene sulphonate (PEDOT:PSS) (Baytron P VP AI4083) as hole injection layer (HIL) was spin coated onto ITO glass (5000 rpm, 30s), followed by baking at 150°C for 15 min. Thereafter, TFB as hole transport layer (HTL) was spin-coated at 1000–4000 rpm, followed by annealing at 130°C for 10 min to achieve different thicknesses. The emissive layer of colloidal ZnCdSeS QDs was spin-coated on HTL (1000 rpm, 30 s) and annealed at 120°C for 30 min. Then a sol-gel ZnO@TiO_2_ solution was deposited on the QDs layer (4000 rpm, 30 s) and annealed at 150°C in air. Finally, a 100 nm aluminum (Al) electrode was thermally evaporated under high vacuum (4 × 10^−6^ Torr) on top of ZnO@TiO_2_ ETL, followed by post-annealing at 100°C for 20 min. The active area of the devices was defined by a shadow mask of 4 mm^2^. The devices are named as sample A, B, C, and D with the TFB layer thickness of 40, 30, 20, and 10 nm, respectively.

### Sample and Device characterization

The films thicknesses were measured by using Filmetrics F20-EXR. The current density-voltage (J-V) characteristics were measured with Keithley-2400 source-meter and electroluminescence (EL) spectra were recorded with an Ocean Optics Maya 2000-PRO spectrometer. The absorption and photoluminescence (PL) spectra were measured U-4100 UV-visible and NIR-300 spectrophotometer, respectively. The structural analysis of the samples was carried out using a Cs-corrected high-resolution transmission electron microscope (HRTEM, Titan 80–300, FEI, U.S.A.) with an information limit 15 of 80 pm operated at an acceleration voltage of 300 kV. The TEM was equipped with a multiscan charge-coupled device (CCD) camera system (Model 894, Gatan, USA) to record the HRTEM images.

## Author Contributions

J.C. and D.W.Z. designed the experiments and wrote the manuscript. J.C performed materials synthesis and W.L. carried out the test of devices. F.X. assisted in the quantum dot characterization. C.L., L.S., A.N. and X.W.S jointly polished the manuscript and gave some comments. All authors reviewed the manuscript and discussed the results.

## Supplementary Material

Supplementary Information Supplementary information
