# Supplementary material for: All Solution-processed Stable White Quantum Dot Light-emitting Diodes with Hybrid ZnO@TiO2 as Blue Emitters
Source: Sci Rep. 2014 Feb 13;4:4085. doi: 10.1038/srep04085 (PMC3923213; doi:10.1038/srep04085)
Supplement: Supplementary Information [file srep04085-s1.doc]

**Supplementary information**

All Solution-processed Stable White Quantum Dot Light-emitting Diodes with Hybrid ZnO@TiO_2_ as Blue Emitters

Jing Chen[[1]](#footnote-2)†, Dewei Zhao^2†^, Chi Li^1^, Feng Xu^1^, Wei Lei1[[2]](#footnote-3)*, Litao Sun^1^, Arokia Nathan^3^ and Xiaowei Sun^4^

^1^School of Electronic Science and Engineering, Southeast University, Nanjing, China, 210096

^2^Department of Electrical Engineering and Computer Science, the University of Michigan, Ann Arbor, Michigan 48109, USA

^3^Electrical Engineering Division, Engineering Department, University of Cambridge, 9 JJ Thomson Avenue, CB3 0FA, Cambridge, UK

^4^School of Electrical and Electronic Engineering, Nanyang Technological University, Nanyang Avenue, Singapore 639798

Figure S1 Surface wetting test for ZnO@TiO_2_ hybrid

Figure S2 AFM image of ZnO@TiO_2_ film

Figure S3The emission track on CIE 1931 chromaticity space of sample C under the driving voltage varying from 10-15 V

Figure S4 Comparison of lifetime of QD-LEDs based on ZnO and ZnO@TiO_2_, respectively.


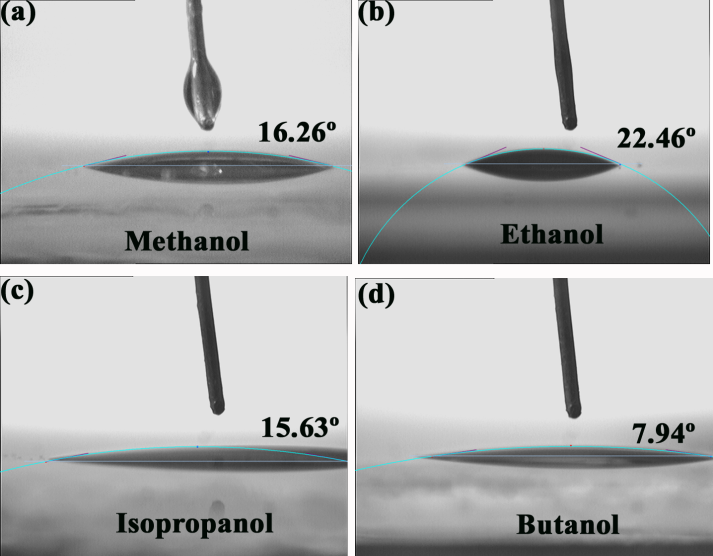


Figure S1 Effect of the solvents used for hybrid ZnO@TiO_2_ dispersed in (a) methanol, (b) ethanol, (c) isopropanol, and (d) butanol, on the surface wettability of QD film. The contact angle is listed in each figure.


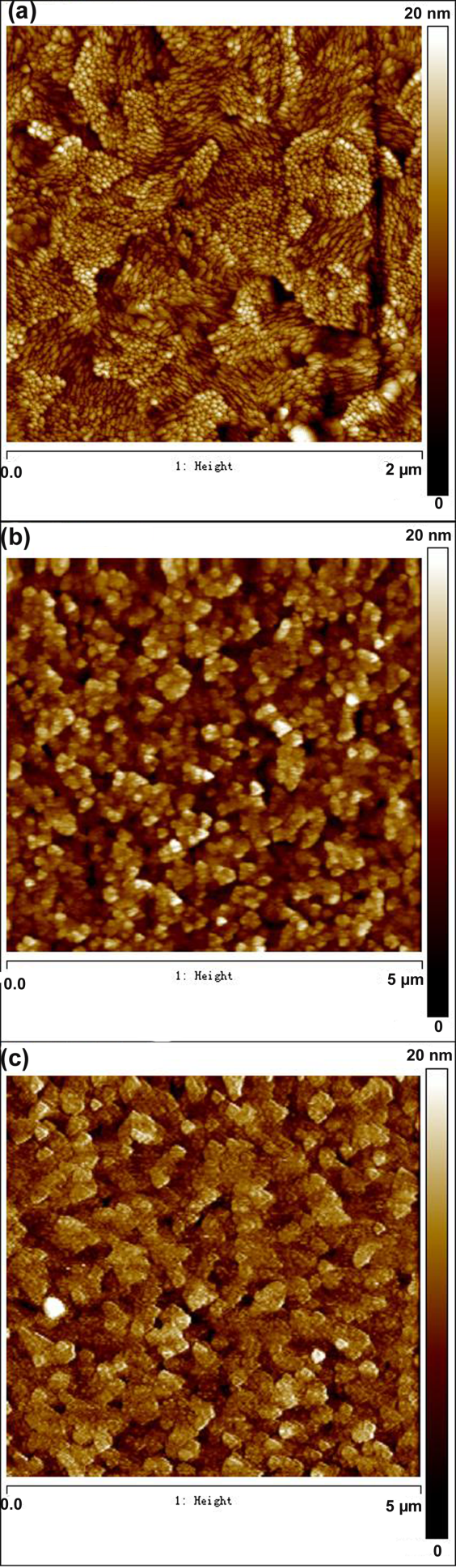


Figure S2 Tapping-mode AFM images of (a) QD (b) ZnO NPs (c) hybrid ZnO@TiO_2_ deposited on QDs layer. The surface roughness (RMS) of QD, ZnO NPs and hybrid ZnO@TiO_2_ is 16, 10 and 3 nm, respectively.


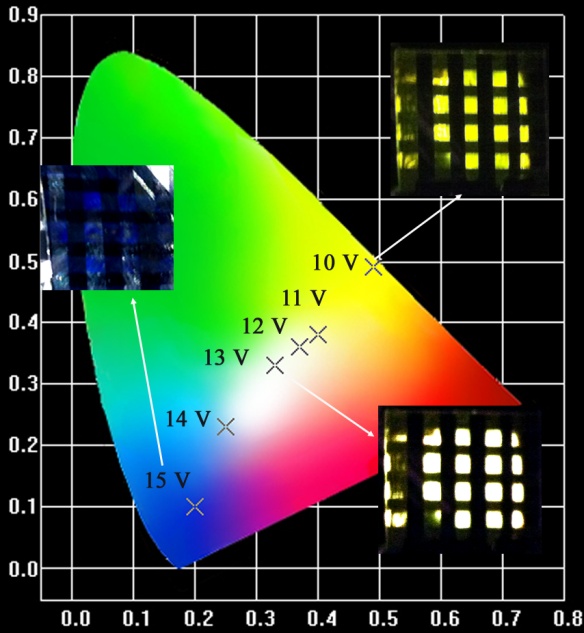


Figure S3 The emission track on CIE 1931 chromaticity space of sample C under the driving voltage varying from 10-15 V with the emitting color changing from yellow light, white light, to blue light. The insets are the photographs of QD-LED devices with yellow, white, and blue light.

Figure S4 Comparison of lifetime of QD-LEDs based on ZnO and ZnO@TiO_2_, respectively. The lifetime of ZnO@TiO_2_ based QD-LED is enhanced, mainly ascribed to the lower current leakage induced by hybrid ZnO@TiO_2_ layer than that by grain boundary of ZnO NPs. In addition, ZnO@TiO_2_ annealed in air as the protect layer prevents the oxygen and moisture from penetrating into the organic layer of QD-LED.

1. ^†^ These authors contributed equally to this work. [↑](#footnote-ref-2)
2. ^*^Corresponding authors: C.J. : [chenjingmoon@gmail.com](mailto:chenjingmoon@gmail.com); W.L. : [lw@seu.edu.cn](mailto:lw@seu.edu.cn) [↑](#footnote-ref-3)
